# Supplementary material for: The Efficiency of Neurospheres Derived from Human Wharton’s Jelly Mesenchymal Stem Cells for Spinal Cord Injury Regeneration in Rats
Source: Int J Mol Sci. 2023 Feb 14;24(4):3846. doi: 10.3390/ijms24043846 (PMC9964265; doi:10.3390/ijms24043846)
Supplement: Supplementary file 1 [file ijms-24-03846-s001.zip › Supplementary Materials_Sirilak_ijms_Final_250123.pdf]

International Journal of *Molecular Sciences*

MDPI

Supplementary Materials

The Efficiency of Neurospheres Derived from Human Wharton's Jelly Mesenchymal Stem Cells for Spinal Cord Injury Regeneration in Rats

Sirilak Somredngan [1], Kasem Theerakittayakorn [1], Hong Thi Nguyen [1,2], Apichart Ngernsoungnern [3], Piyada Ngernsoungnern [3], Pishyaporn Sritangos [3], Mariena Ketudat-Cairns [4], Sumeth Imsoonthornruksa [4], Nattawut Keeratibharat [5], Rangsirat Wongsan [6], Ruttachuk Rungsiwiwut [7] and Rangsun Parnpai [1,*]

1 Embryo Technology and Stem Cell Research Center, School of Biotechnology, Institute of Agricultural Technology, Suranaree University of Technology, Nakhon Ratchasima 30000, Thailand
2 Laboratory of Embryo Technology, Institute of Biotechnology, Vietnam Academy of Science and Technology, Hanoi 100000, Vietnam
3 School of Preclinical Sciences, Institute of Science, Suranaree University of Technology, Nakhon Ratchasima 30000, Thailand
4 School of Biotechnology, Institute of Agricultural Technology, Suranaree University of Technology, Nakhon Ratchasima 30000, Thailand
5 School of Surgery, Institute of Medicine, Suranaree University of Technology, Nakhon Ratchasima 30000, Thailand
6 The Center for Scientific and Technological Equipment, Suranaree University of Technology, Nakhon Ratchasima 30000, Thailand
7 Department of Anatomy, Faculty of Medicine, Srinakharinwirot University, Bangkok 10000, Thailand
* Correspondence: rangsun@g.sut.ac.th; Tel.: +66-442-242-34

Contents

Figure S1. Number of spheres after filtration (diameter 100-140 μm).

Figure S2. Western blot analysis. (A) Western blot images of NeuroD1 protein, β-actin protein was used as an internal control. (B) Quantification of western blot results. Data were shown as mean ± S.D.

Table S1. Antibodies used for flow cytometry and immunofluorescent.

Table S2. Primers used for qPCR.

Supplementary Figures

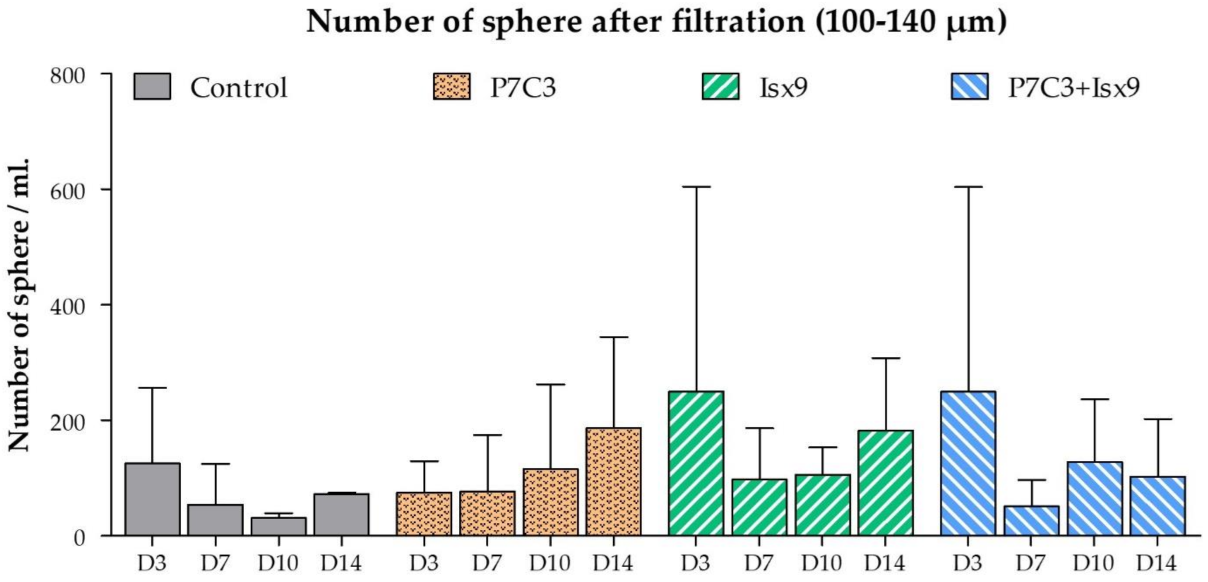

Figure S1. Number of spheres after filtration (diameter 100-140 μm).

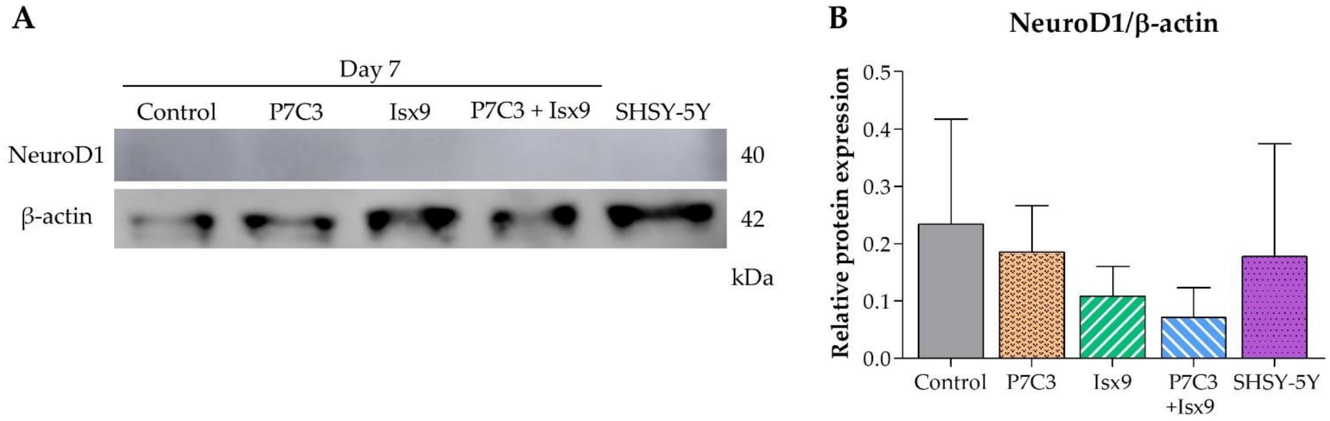

Figure S2. Western blot analysis. (A) Western blot images of NeuroD1 protein, β-actin protein was used as an internal control. (B) Quantification of western blot results. Data were shown as mean ± S.D.

Supplementary Tables

Table S1. Antibodies used for flow cytometry and immunofluorescent.

Antibodies	Companies	Cat #
APC mouse anti-CD73	BioLegend	344006
APC/Cy7 mouse anti-CD90	BioLegend	328132
PE mouse anti-CD105	BioLegend	323206
PE mouse anti-CD34	Beckman Coulter	A07776
FITC mouse anti-CD45	BioLegend	368508
FITC mouse IgG, isotype Ctrl	BioLegend	400109
APC mouse IgG, isotype Ctrl	BioLegend	400120
PE mouse IgG, isotype Ctrl	BioLegend	400113
Mouse anti-nestin	Merck	MAB5326
Mouse anti-β-tubulin 3	Merck	MAB1637
Rat anti-SOX2	eBioscience	14-9811-82
Mouse anti-NeuroD1	Merck	WH0004760M1
Mouse anti-DCX	Merck	MABN707
Rabbit anti-β-catenin	Merck	06-734
Cy3 mouse anti-nuclei	Merck	MAB1281C3
Goat anti-ChAT	Merck	AB144P
Mouse anti-MAP2	Merck	MAB3418
Mouse anti-GFAP	BioLegend	644702
Mouse anti-NF-L	Merck	MAB1615
Mouse anti-Olig2	Merck	MABN50
Rabbit anti-BDNF	Merck	AB1534SP
Mouse anti-β-actin	Affinity Biosciences	T0022
Goat anti-mouse IgG HRP	Abcam	AB6789

Table S2. Primers used for qPCR.

Genes	Primer sequence (5'→3')	Product size (bp)	References
β-catenin	F: CTGAGGACAAGCCACAAGATTACA<br>R: TGGGCACCAATATCAAGTCCAA	121	NM 001330729.2
β-tubulin 3	F: TGGATCCCCAACAACGTGAA<br>R: CITCGTACATCTCCCCTCTT	286	NM 006086.4
NeuroD1	F: TCTTCCACGTTAAGCCTCCG<br>R: CCATCAAAGGAAGGGCTGGT	97	NM 002500.5
SOX2	F: GCGGAAAACCAAGACGCTC<br>R: TTCATGTGCGCGTAACTGTC	153	BC 013923.2
nestin	F: AGTGATGCCCCTTCACCTTG<br>R: GCTCGCTCTCTACTITCCCC	199	NM 006617.1
DCX	F: TATGCGCCGAAGCAAGTCTCCA<br>R: CATCCAAGGACAGAGGCAGGTA	155	NM 000555.3
*β-actin**	F: GAGAAAATCTGGCACCACACC<br>R: GGATAGCACAGCCTGGATAGCAA	177	NM 001101.5

*Reference gene
